# Quality of work life of rural emergency department nurses and physicians: a pilot study

**DOI:** 10.1186/s13104-015-1075-2

**Published:** 2015-04-01

**Authors:** Isabelle Bragard, Richard Fleet, Anne-Marie Etienne, Patrick Archambault, France Légaré, Jean-Marc Chauny, Jean-Frédéric Lévesque, Mathieu Ouimet, Julien Poitras, Gilles Dupuis

**Affiliations:** Health Psychology Unit, Liège, Université de Liège, Liège, Belgium; Department of Family and Emergency Medicine, Research Centre of the Hôtel-Dieu de Lévis Hospital, Université Laval, Lévis, Canada; Department of Family and Emergency Medicine, Knowledge Transfer and Health Technology Assessment of the CHUQ Research Centre (CRCHUQ), Unité de Recherche Evaluative, Université Laval, Quebec City, Canada; Direction des Systèmes de soins et services, Institut national de santé publique du Québec, Montréal, Québec Canada; Département de science politique, Pavillon Charles-De Koninck, Université Laval, Quebec City, Canada; Department of Psychology, Université du Québec à Montréal, Montreal, QC Canada; Centre de liaison sur l’intervention et la prévention psychosociales (CLIPP), Montreal, QC Canada; Research Centre of the Hôtel-Dieu de Lévis Hospital, Université Laval, 143 Wolfe Street, Lévis, QC G6V 3Z1 Canada

**Keywords:** Quality of work life, Emergency department, Nurses, Physicians, Rural area

## Abstract

**Background:**

Information about recruitment and retention factors and quality of work life (QWL) in rural emergency departments (EDs) is limited. A pilot study was used to determine the feasibility of a large-scale study of these variables in Quebec EDs.

**Methods:**

Two EDs, approximately 10,000 and 30,000 patients per year respectively, were selected as convenience samples. An online survey containing the Quality of Work Life Systemic Inventory (QWLSI; 34 items) and the Recruitment and Retention Factors Questionnaire (39 items) was sent to ED nurses and physicians of these two EDs. Descriptive statistics of percentage, mean and standard deviation and correlations were used to analyse the data.

**Results:**

Forty out of 64 eligible workers (62%) gave their consent to participate, but only 20 had completed both questionnaires. Participants’ mean age was 42 years (SD = 11.6). The average participants satisfaction with their access to continuing education was low (Mean = 1.6, SD = 0.8). However, their satisfaction with technical resources (Mean = 2.4, SD = 0.7), pre-hospital and inter-hospital transfer services (Mean = 2.5, SD = 0.6), relationships with colleagues (Mean = 2.7, SD = 0.6) and managers (Mean = 2.2, SD = 0.7), work-life balance (Mean = 2.4, SD = 0.6) and emergency patient access to other departments (Mean = 3.7, SD = 0.6) was in the average. The impact of several aspects of the rural environment (*e.g.* tranquility) on quality of life was also in the average (Mean = 2.5, SD = 0.7). QWL was in the average, excepted subscale ‘support offered to employee’ for which the QWL was lower.

**Conclusions:**

Data collection was difficult and the larger study will require strategies to improve recruitment such as a paper alternative. The study showed globally good recruitment and retention factors and QWL for these ED nurses and physicians. These results will help hospital administrations better plan initiatives aimed at improving retention and QWL.

## Background

Emergency departments (EDs) are particularly stressful work environments. This can be explained by difficult work conditions including significant workload and psychological demands, lack of resources, and poor support [1-3]. In consequence, ED nurses and physicians showed moderate to high levels of burnout [1,2]. However, the majority of research on stress in EDs is conducted in urban settings.

Even so, in comparison to their urban counterparts, nurses and physicians in rural settings face more challenging working conditions. In addition to the difficult working conditions common to urban settings, specific challenges to rural areas include limited access to specialized care [4], geographical distance from specialized centers, poor emergency transport capabilities [5], and limited training [6]. Rural healthcare facilities also face chronic problems with staff recruitment and retention [6]. In fact, almost every country reports shortages of health professionals in rural areas [7]. This shortage could increase the workload of regular staff, negatively affecting morale and making healthy lifestyles difficult to achieve. Working in such conditions is likely to contribute to burnout and to poor quality of work life (QWL) in rural ED nurses and physicians. Policy-makers need evidence that would allow them to identify which factors could increase the recruitment and retention of nurses and physicians practicing in rural areas [7,8]. It is important to assess their particular difficulties, as rural EDs constitute a safety net of sorts for the 20% of Canadians who live in rural areas [9]. However, to our knowledge, no studies to date have explored factors favoring recruitment and retention, QWL in rural ED nurses and physicians and the associations between these factors.

The study described here constitutes a preliminary step in a larger study designed to develop a portrait of all EDs in rural Quebec [10,11]. This pilot study had two objectives. The first one was to assess the feasibility of conducting an extensive evaluation of recruitment and retention factors and QWL in ED nurses and physicians in Quebec, by assessing the participation rate and the difficulties in data collection of ED nurses and physicians in two rural EDs in Quebec. The second objective was to analyze preliminary data concerning recruitment and retention factors and QWL in these two rural EDs and the associations between these factors.

## Methods

### Study design

This was a cross-sectional study utilizing a web-based survey.

### Setting

The study was conducted in Quebec, a province in east-central Canada occupying a territory nearly three times the size of France or Texas. Most of which is very sparsely populated. It had a population density of 5.8/km2. There are twenty-six emergency departments in Quebec, matching the Statistics Canada criteria for a rural ED [12,13]. Rural EDs are facilities offering 24/7 medical coverage, having hospitalization beds and located in a rural or small town. These EDs had an average capacity of 41 ± 20 acute-care beds and 36 ± 43 long-term beds, and 19321 ± 6275 annual ED patient visits. Among these 26 EDs, a convenience sample of two EDs was selected and assessed between May 1 and July 15, 2013. The two EDs were chosen because they were representative of different infrastructure size (one small and one large center), of different levels of equipment and different regions of Quebec. The first ED receives approximately 10,000 patients per year (45 beds); the second receives approximately 30,000 patients per year (70 beds).

### Population

The first ED included 20 nurses and 9 physicians, and the second ED included 20 nurses and 15 physicians.

### Instrument

The online survey had three parts. The first part included items concerning personal and professional variables such as age or work schedule. The second part evaluated recruitment and retention factors, and was developed specifically for this study. Item development was inspired by clinical experience with recruitment and retention in rural EDs, as well as by an unpublished literature review on the topic. This contained 39 items gathered from existing literature on recruitment and retention and on factors influencing recruitment and retention. Those included continuing education, emergency patient access to other departments (*e.g.*, oncology), work satisfaction with several resources (*e.g.*, relationships with colleagues), and the impact of several aspects of the rural environment on quality of life (*e.g.*, work climate). The factors were primarily assessed on a 5-point Likert scale ranging from ‘not at all’ (0) to ‘extremely’ (4), excepted for ‘emergency patient access to other departments’ on a 6-point Likert scale ranging from ‘not available’ (0) to ‘excellent’ (5) (the minimum score was 0 with a maximum score of 167).

**Figure 1:**
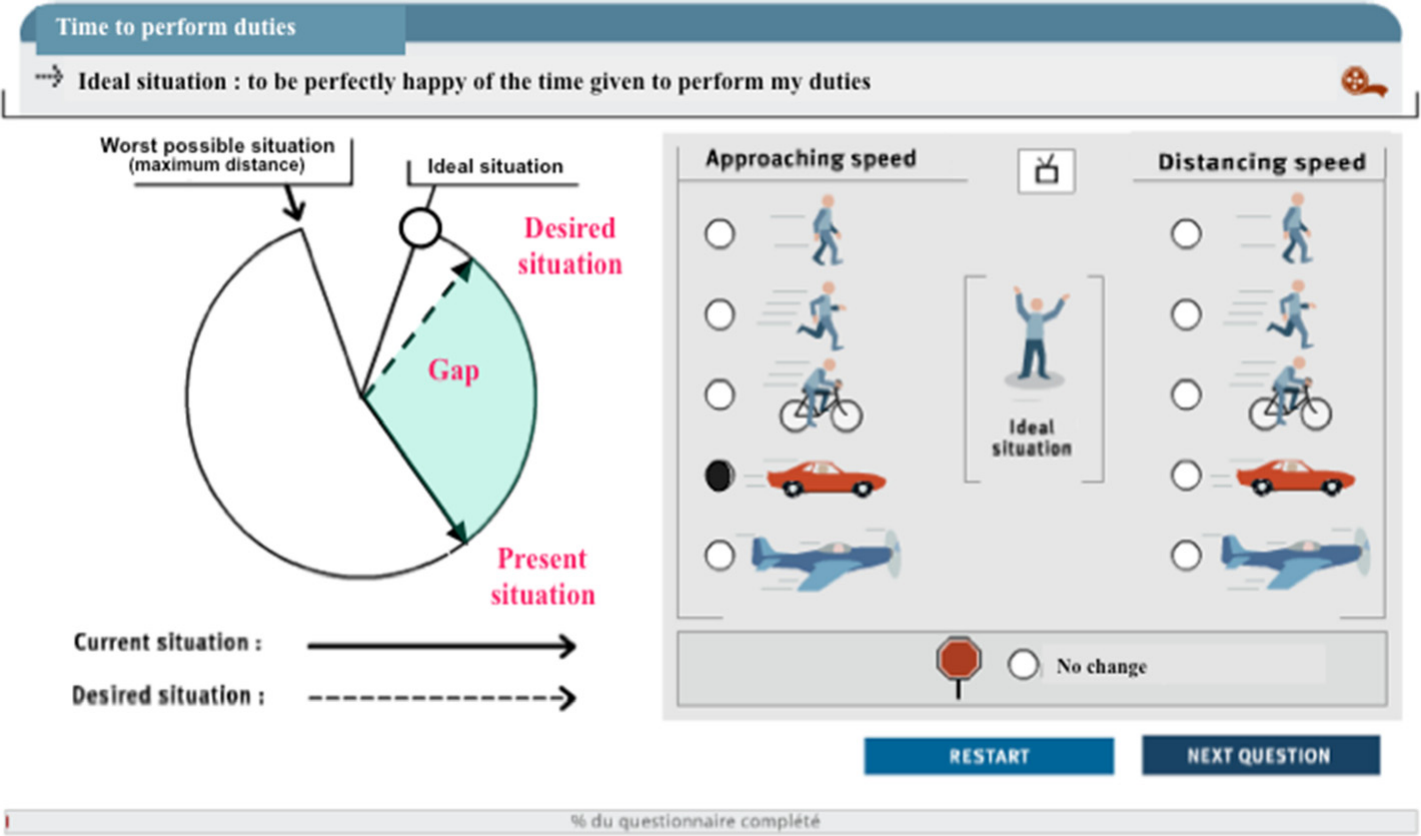
**Item example of Quality of Work Life Systemic Inventory (QWLSI).** Each item of the QWLSI is measured using a Visual Analogue Scale-type dial. One side of the circle represents the ideal situation; the other side represents the worst possible situation. Participants use arrows to indicate the location of the current status and of a status they would consider satisfactory, relative to a predetermined ideal. Next, in the box to the right of the figure, participants indicate the extent to which conditions are improving or deteriorating, and at what rate.

The third part was the Quality of Work Life Systemic Inventory (QWLSI) [14] and was available via http://qualitedevie.ca. This covered 34 items divided into eight subscales: compensation, career growth, work schedule, relationship with colleagues, relationship with superiors, physical environment, factors influencing appreciation of tasks, and employee support. Based on a literature search and clinical experience, we included an additional module containing six items designed to capture aspects specific to EDs that were not addressed by the existing questionnaire items: 1) relationship with patients, 2) relationship with patients’ families, 3) nature of emergency patients’ presenting problems, 4) emotional support from colleagues following a stressful/traumatic experience, 5) evolution of the profession, and 6) evolution of working conditions. The QWLSI takes approximately 30–40 minutes to complete. Each item is measured using a Visual Analogue Scale-type dial (see Figure 1). One side of the circle represents the ideal situation; the other side represents the worst possible situation. Participants use arrows to indicate the location of the current state and of a state they would consider satisfactory (goal), relative to a predetermined ideal. Next, in the box to the right of the figure, participants indicate the extent to which conditions are improving or deteriorating, and at what speed. Finally, the importance of each item is rated on a Likert scale from 1 (essential to my life) to 7 (completely useless). There are two ways to analyze QWLSI. First, the QWLSI could yield three global scores: gap, goal, and rank. The gap score corresponds to QWL, and is calculated as the mean distance between the current state and the goal state for each item, weighted by speed of improvement or deterioration, and the item’s rank. The gap score varies from −100 (minimum) to 100 (maximum). *Higher scores represent poorer QWL*. The goal score represents the mean distance between the desired situation (goal) and the ideal situation, and provides information about the desired level of happiness. The goal score also varies from 0 (minimum) to 100 (maximum). *Higher scores indicate lower goals* (i.e., goals that are further from the ideal). The rank score is the mean ranking for the 34 items, and reflects the priority assigned to the respective area of work life. For the rank, the minimum score is 0.12 with a maximum of 2. *Higher scores represent higher importance of the domain* (see Martel et al. [14] for further details about calculation). The second way to analyze QWLSI data is based on the conversion of gap scores to percentiles based on comparison to a database of scores from over 3,500 workers in various professions (*e.g.*, health care professionals, managers) from Canada, Belgium, and Switzerland. A percentile indicates the value below which a given percentage of observations in a group of observations falls. Scores above the 50th percentile indicate good QWL, scores from the 25th to 49th percentile indicate areas where improvement is desired, and scores below the 25th percentile indicate problem areas (greater psychological distress and professional burnout) [15]. Global consistency (Cronbach’s alpha) for QWLSI is 0.87; subscale consistency ranges from 0.60 to 0.82, and test-retest reliability is 0.84 (*p* < 0.001) [16].

### Procedure

The medical directors and head nurses of the two respective EDs were contacted and provided information about the study and its objectives. They were asked to present the project to all ED nurses and physicians, and to complete the consent forms for their center. Once consent for the study was given, nurses and physicians could provide further consent to receive an email invitation to complete the online survey. No financial incentive was offered.

#### Outcomes

The primary outcome measure was feasibility of the study. Investigators had pre-established criteria for determining feasibility: 1) participation rate of 50% or greater among health professionals solicited for participation, and 2) survey completion rate of 70% or greater among health professionals who consented to participate. These criteria were an educated guess based on achieving a representative sample. Since the study was designed to determine feasibility only, no sample size calculation was performed (as per Légaré et al. [17]). Secondary outcomes were preliminary responses concerning recruitment and retention factors and QWL.

### Data analysis

Descriptive statistics (means and standard deviation (SD)) were used to describe the proportions of workers who completed the online survey and to examine their personal and professional data, retention and recruitment factors and QWLSI results. Then, inferential statistics (Pearsons correlations) were computed to assess the association between retention and recruitment factors and quality of work life. The analyses were performed with SPSS V.13 (SPSS Inc. Chicago Illinois) [18].

### Ethics

The study was approved by the Alphonse-Desjardins’ Center for Health and Social Services ethics review board (Project MP-HDL-1213-011), and each participant provided consent to participate.

## Results

### Personal and professional data

The 20 participants included 17 nurses and three physicians, and 15 women and five men (See Table 1). The majority of participants were born in rural areas (*n* = 16; 80%); mean age was 42 years (SD = 11.6; range 25–60). The majority of participants had over six years of work experience (*n* = 17; 85%). Two physicians were trained family doctors, and one had completed training/certification in emergency medicine. Nine nurses had college degrees, and eight nurses had university degrees. The majority of participants worked between 31 and 40 hours per week (*n* = 13; 65%).

**Table 1:** **Sociodemographic and professional data**

**Variables**	***n***	**%**
Gender		
Male	5	25
Female	15	75
Institution		
ED1	8	40
ED2	12	60
Profession		
Nurse	17	85
Physician	3	15
Age (years)		
18-30	5	25
31-40	6	30
41-50	3	15
51-60	6	30
Education		
Less than 7 years	1	5
7-11 years	6	30
12-15 years	13	65
Number of years work experience		
Less than 1 year	1	5
1-5 years	2	10
6-10 years	5	25
11-20 years	5	25
Over 21 years	7	35
Number of years in this ED		
Less than 1 year	2	10
1-5 years	4	20
6-10 years	4	20
11-20 years	4	20
Over 21 years	6	30
Originally from a rural area	16	80
Income		
$30,001 to $50,000	2	10
$50,001 to $70,000	5	25
$70,001 to $100,000	10	50
More than $100,000	3	15
Work schedule		
Number of hours a week (including overtime)		
11-20 hours	2	10
21-30 hours	3	15
31-40 hours	13	65
More than 41 hours	2	10
Frequency of overtime (never 0 – always 4)		
Never	5	25
Rarely	3	15
Sometimes	10	50
Often	2	10
Always	0	0

### Recruitment and participation (feasibility)

Forty of 64 eligible nurses and physicians (62.5%) consented to participate in the pilot study and signed the consent form. After they provided consent, participants received up to three email reminders to complete the survey. Three additional strategies designed to boost response rate were implemented: a follow-up telephone call to medical directors and head nurses, posters describing the project posted in the participating EDs, and distribution of a video of two of the primary investigators (RF and GD) encouraging participation. Only 20 of the 40 consenting professionals had completed both questionnaires (50%). Eight additional participants had completed the QWLSI only (28/40, 70%).

### Recruitment and retention factors

The average participants satisfaction with their access to continuing education was low (Mean = 1.6, SD = 0.8) (See Table 2). However, their satisfaction with emergency patient access to other departments (oncology, cardiology, anesthesiology, mental health, operating theater, intensive care, diagnostic services, advanced diagnostic services, drugs and medical devices, family physicians) (Mean = 3.7, SD = 0.6), with technical resources (Mean = 2.4, SD = 0.7), with pre-hospital and inter-hospital transfer services (Mean = 2.5, SD = 0.6), with relationships with colleagues (Mean = 2.7, SD = 0.6), with relationships with managers (Mean = 2.2, SD = 0.7), and with balance between personal and professional commitments (Mean = 2.4, SD = 0.6) was in the average. The impact of several aspects of the rural environment on quality of life was also in the average: aesthetic qualities (Mean = 2.5, SD = 0.7), good weather (Mean = 2.5, SD = 0.8), tranquility (Mean = 2.7, SD = 0.7), presence of different services (Mean = 2.1, SD = 0.7) and the advantageous cost of living (Mean = 2.1, SD = 0.9).

**Table 2:** **Recruitment and retention factors**

**Variables**	**n**	**%**	**Mean**	**SD**
Satisfaction with access to continuing education (0 to 4)			1.6	0.8
Not at all	1	5.3		
A little	8	42.1		
Moderately	8	42.1		
Very	2	10.5		
Extremely	0	0		
Patient access to other departments (*e.g.*, oncology) (0 to 5)			3.7	0.6
Not available	0	0		
Poor	0	0		
Fair	0	0		
Good	5	31.2		
Very good	9	56.3		
Excellent	2	12.5		
Work satisfaction (0 to 4)				
Technical resources			2.4	0.7
Not at all	0	0		
A little	1	5		
Moderately	12	60		
Very	6	30		
Extremely	1	5		
Pre-hospital and inter-hospital transfers services			2.5	0.6
Not at all	0	0		
A little	1	5		
Moderately	9	45		
Very	10	50		
Extremely	0	0		
Relationships with colleagues			2.7	0.6
Not at all	0	0		
A little	0	0		
Moderately	7	35		
Very	12	60		
Extremely	1	5		
Relationships with managers			2.2	0.7
Not at all	0	0		
A little	3	15		
Moderately	11	55		
Very	6	30		
Extremely	0	0		
Balance between personal and professional commitments			2.4	0.6
Not at all	0	0		
A little	1	5		
Moderately	10	50		
Very	9	45		
Extremely	0	0		
Quality of life: positive impact of (0 to 4)				
Aesthetic qualities of the environment			2.5	0.7
Not at all	0	0		
A little	2	10		
Moderately	6	30		
Very	12	60		
Extremely	0	0		
Presence of a good weather			2.5	0.8
Not at all	1	5		
A little	1	5		
Moderately	6	30		
Very	12	60		
Extremely	0	0		
Presence of a tranquility of the environment			2.7	0.7
Not at all	0	0		
A little	5	5		
Moderately	25	25		
Very	65	65		
Extremely	5	5		
Presence of different activities and services (sports and recreational activities, cultural activities, shopping, good schools)			2.1	0.7
Not at all	0	0		
A little	3	15		
Moderately	8	40		
Very	9	45		
Extremely	0	0		
Presence of advantageous cost living (*e.g.*, property prices)			2.1	0.9
Not at all	2	10		
A little	2	10		
Moderately	6	30		
Very	10	50		
Extremely	0	0		
Factors most frequently cited as contributing to QOL				
Tranquility of the environment	16	80		
Aesthetic qualities of the environment	8	40		
Access to good jobs for spouse/partner	4	20		
Access to sport and recreational activities	4	20		

### Quality of work life

The analysis of QWLSI results was conducted in two steps: 1) analysis of the eight subscale scores (gap, goal and rank), and 2) analysis of the gap scores converted in percentiles. First ED workers had a main goal score of 19.6 on average (SD = 8.6) (see Table 3). The goal subscale scores were between 14.5 and 20.2. Their goals are quite close to the ideal. They had a main gap score of 5 (SD = 4.7), which is in the average. The gap subscale scores were between 3.7 and 7.4. One gap subscale score ‘support offered to employee’ was higher, meaning lower quality of work life in this domain (Mean = 7.4; SD = 10.9). They had a main rank score of 1.6 (SD = 0.2). The rank subscale scores were between 1.3 and 1.7. All the domains have a high priority level, showing that they find it difficult to rank them. The subscale ‘support offered to employee’ showed high gap score and high priority.

**Table 3:** **Global scores and subscale scores of quality of work life (n = 28)**

**Quality of work life inventory**	**Mean (SD)**
Main scores	
Goal	19.6 (8.6)
Gap	5.0 (4.7)
Range	1.6 (0.2)
Subscale scores	
Goal	
Compensation and benefits	15.3 (6.4)
Career path	20.2 (9.1)
Arrangement of work schedule	18.0 (6.2)
Climate with colleagues	17.4 (6.8)
Climate with superiors	19.5 (9.9)
Characteristics of physical environment related to task	14.5 (7.2)
Factors influencing appreciation of tasks to be done	17.0 (5.0)
Support offered to employee	19.5 (6.8)
Specific ED items	16.9 (11.2)
Gap	
Compensation and benefits	3.8 (3.5)
Career path	6.3 (6.7)
Arrangement of work schedule	6.1 (8.8)
Working relationship with colleagues	3.5 (4.0)
Working relationship with superiors	6.1 (7.7)
Characteristics of physical environment related to task	5.0 (9.0)
Factors influencing appreciation of tasks to be done	3.7 (3.5)
Support offered to employee	7.4 (10.9)
Specific ED items	4.5 (4.4)
Rank	
Compensation and benefits	1.7 (0.2)
Career path	1.4 (0.4)
Arrangement of work schedule	1.7 (0.3)
Working relationship with colleagues	1.6 (0.4)
Working relationship with superiors	1.6 (0.3)
Characteristics of physical environment related to task	1.4 (0.3)
Factors influencing appreciation of tasks to be done	1.6 (0.2)
Support offered to employee	1.3 (0.4)
Specific ED items	1.6 (0.6)

Second, when the gap scores were converted to percentiles, the global QWL was at the 50th percentile, confirming that QWL in our sample falls within the average range. However, independent analysis of each item indicated that QWL was below the 25th percentile in six areas: competitiveness, physical nature of the workload, relationships with employees, family leave policy, support facilities (*e.g.*, child care, parking) and working conditions (human and material resources). This last item comes from the additional specific module. Family leave policy and support facilities come from the subscale ‘support to employee’. QWL was reported to be good (>50th percentile) for all other domains (relationships with patients, relationship with patients’ families, patients’ presenting problems, evolution of the profession, and emotional support from colleagues after a stressful/traumatic experience).

### Associations between retention and recruitment factors and QWL

Three variables were significantly correlated with global QWL: satisfaction about technical resources (r = −0.42, p = 0.066), satisfaction about pre-hospital and inter-hospital transfers services (r = −0.54, p = 0.015), and satisfaction about relationships with managers (r = −0.49, p = 0.028). So a high QWL was associated with a high satisfaction about these resources.

## Discussion

The results of this pilot study indicate that conducting a larger trial to assess recruitment and retention factors and QWL in rural ED nurses and physicians is feasible. Only twenty of the 40 individuals who consented to participate have completed the two questionnaires. The results revealed that while their satisfaction with access to continuing education was low, their satisfaction with patient access to other departments, and with patient access to technical resources and to pre-hospital and inter-hospital transfer services was in the average. Global QWL was also in the average, excepted for ‘support offered to employee’ for which the QWL was lower. These results lead us to three primary conclusions.

First, the data collection was difficult. The number of people willing to participate in the study (signed consent) was in the average (40/64, 62%) compared to the rate of 80% considered as high in the literature [19]. But, only 20 out of 40 had completed the retention and recruitment factors questionnaire (RRFQ) compared to 28 out of 40 for the previously validated QWLSI. As a newly developed questionnaire, the RRFQ could be a potential weakness to optimizing participation in the larger study. Some of the questions (*e.g.*, the positive impact of several characteristics of the rural environment on quality of life) could be removed to shorten the time taken to complete the survey, because they could be redundant. Moreover, several response rate factors must be taken into consideration. The average response rate to email surveys in scientific studies appears to be decreasing, whereas the number of this type of study has been increasing [20]. Moreover, healthcare professionals may have had difficulty completing the questionnaires for several reasons, including limited access to a computer at work, difficulty accessing the questionnaires (the two questionnaires were not linked together, but were available on two different platforms), difficulty using the QWLSI website, questionnaire length (30–40 minutes for QWLSI), and limited available time to complete the surveys. Lovato et al. have already demonstrated that investigators often underestimate the costs and time needed for recruitment, and often require extensions or significant effort to meet recruitment targets [21]. In fact, length of survey has been considered to have a negative influence on survey response rates in other studies [22]. In the event of a larger study, methods of improving recruitment must be implemented. We propose to link the two questionnaires together in one platform, to train a local leader in each ED on the use of the QWLSI website, to give didactic materials in the form of a video tutorial and online instructions, to offer the paper alternative, and to negotiate with ED heads for employees to have a dedicated time to complete the questionnaires. The use of tablet computers could also save time and make the questionnaires more user-friendly [23]. Finally, financial rewards or prizes have been proven to be an effective incentive for participation in studies [24,25].

Where retention and recruitment factors are concerned, nurses and physicians reported dissatisfaction with access to continuing education. Studies have demonstrated that ongoing training was an important retention factor for health professionals in under-served areas (*e.g.* [26]). Providing training opportunities could improve ED professionals’ knowledge, skills, and self-confidence, subsequently reducing work-related stress [27]. In fact, systematic reviews that addressed quality improvement and continuing education strategies demonstrated that providing training opportunities yielded a 10% improvement in performance [28,29]. In contrast to their dissatisfaction with training opportunities, nurses and physicians in the participating EDs were quite satisfied with the locally available of resources. This finding is inconsistent with others studies that reported limited resources in rural centers [4,30-35]. However, the limited number of participants in this study (particularly the limited number of physicians) precludes meaningful interpretation of this finding.

Third, ED nurses and physicians reported QWL in the average. They were motivated to reach their goals. However, they find it difficult to rank the several domains. This means that they may experience more tension when they have to allocate their time to one area instead of another because every area has the same level of importance. Moreover, their QWL was lower for six items, two (family leave policy and support facilities) belong to the subscale ‘support offered to employees’. ED workers also showed a low QWT for the specific domain of ED working conditions (human and material resources). These domains could constitute psychosocial risk factors, which suggests the need for interventions. Promising strategies for professional and personal support [7] include material support such as child care and accommodations; such material support has been demonstrated to encourage rural practice [26]. Peer support, increased social support from superiors and culture of openness and tolerance could also help to overcome difficult working conditions and to avoid isolation [36].

Finally, the correlation results have suggested that when satisfaction about technical resources, about pre-hospital and inter-hospital transfers services and about relationships with managers were low, the global QWL was low. The presence of these associations should be checked in the larger study. If confirmed, these factors could also be the target of interventions to increase the QWT.

Several limitations of this study must be acknowledged. First, the pilot study was restricted to two rural EDs. Participation of four or five EDs would have created a more representative sample. Indeed, the representativeness of the sample seems low. The majority of this sample had worked in these EDs for six years or more. This seems very high, particularly when compared to a recent Australian study indicating that only about 20% of rural doctors remained in their practice for six years or more [37]. Second, the number of doctors participating in the pilot study was quite low (3/20). It did not permit comparison of nurses and physicians data, potentially obscuring differences in satisfaction between the two professions. The above ideas to improve recruitment will undoubtedly increase the number of doctors in the larger study. Third, there may have been differences between participating and non-participating ED nurses and physicians, i.e., nurses and physicians who participated may have had greater personal interest or motivation for the study relative to non-participants.

## Conclusions

The results allowed us to conclude that the ED nurses and physicians in our study had overall good QWL, and allowed us to identify targets for potential interventions. However, the results also confirm that recruitment for this type of study is difficult and that a larger study will require strategies to improve recruitment. The results of the larger study will yield a greater understanding of the factors associated with work-related quality of life in ED professionals, and of the factors associated with recruitment and retention of ED personnel.

## Abbreviations

ED: Emergency department
QWL: Quality of work life
QWLSI: Quality of Work Life Systemic Inventory

## References

[1] Potter C (2006). To what extent do nurses and physicians working within the emergency department experience burnout: a review of the literature. Australas Emerg Nurs J.

[2] Bragard I, Dupuis G, Fleet R. Quality of work life, burnout, and stress in emergency department physicians: a qualitative review. Eur J Emerg Med 2014 Aug 2. [Epub ahead of print]. 10.1097/MEJ.0000000000000194 25093897

[3] Josland H, Dolan B, Holt L (2008). Stress and stress management. Accident and emergency: theory into practice.

[4] Haggerty JL, Roberge D, Pineault R, Larouche D, Touati N (2007). Features of primary healthcare clinics associated with patients’ utilization of emergency rooms: urban–rural differences. Healthc Policy.

[5] Carr BG, Caplan JM, Pryor JP, Branas CC (2006). A meta-analysis of prehospital care times for trauma. Prehosp Emerg Care.

[6] Canadian Association of Emergency Physicians (1997). Recommendations for the management of rural, remote and isolated emergency health care facilities in Canada.

[7] Grobler L, Marais BJ, Mabunda SA, Marindi PN, Reuter H, Volmink J. Interventions for increasing the proportion of health professionals practising in rural and other underserved areas. Cochrane Database Syst Rev, 2009; Issue 1. Art. No.: CD005314. doi: 10.1002/14651858.CD005314.pub2. 10.1002/14651858.CD005314.pub2 19160251

[8] Chopra M, Munro S, Lavis JN, Vist G, Bennett S (2008). Effects of policy options for human resources for health: an analysis of systematic reviews. Lancet.

[9] DesMeules M, Pong R. Comment se portent les Canadiens vivant en milieu rural? Une évaluation de leur état de santé et des déterminants de la santé. In: Canada AdlSPd editor. Ottawa: Institut canadien d’information sur la santé; 2006.

[10] Fleet R, Archambault P, Legare F, Chauny JM, Levesque JF, Ouimet M (2013). Portrait of rural emergency departments in Quebec and utilisation of the Quebec Emergency Department Management Guide: a study protocol. BMJ Open.

[11] Feeley N, Cossette S, Cote J, Heon M, Stremler R, Martorella G (2009). The importance of piloting an RCT intervention. Can J Nurs Res.

[12] du Plessis V, Beshiri R, Bollman RD, Clemenson H (2002). Definitions of “rural”. Agriculture and rural working paper series working paper.

[13] Association canadienne des soins de santé. Guide des établissements de soins de santé du Canada. Ottawa: CHA, 2009–2010.

[14] Martel JP, Dupuis G (2006). Quality of work life: theoretical and methodological problems, and presentation of a new model and measuring. Soc Indic Res.

[15] Bragard I, Dupuis G, Razavi D, Reynaert C, Etienne AM (2012). Quality of work life in doctors working with cancer patients. Occup Med (Lond).

[16] Dupuis G, Martel J. Caractéristiques psychométriques de l’ISQVT©: Fidélité test-retest, cohérence interne, validité de construit, validation transculturelle: In: Battistelli A, Depolo M, Fraccaroli F, editors. La qualité de la vie au travail dans les années 2000. Actes du 13e Congrès de Psychologie du Travail et des Organisations. CD-ROM. Bologna: CLUEB; pp.699–706.

[17] Legare F, Labrecque M, Leblanc A, Thivierge R, Godin G, Laurier C (2007). Does training family physicians in shared decision making promote optimal use of antibiotics for acute respiratory infections? Study protocol of a pilot clustered randomised controlled trial. BMC Fam Pract.

[18] SPSS (2004). SPSS base 13.0 for windows user’ s guide.

[19] Choung RS, Locke GR, Schleck CD, Ziegenfuss JY, Beebe TJ, Zinsmeister AR (2013). A low response rate does not necessarily indicate non-response bias in gastroenterology survey research: a population-based study. J Public Health.

[20] Sheehan K (2001). E-mail survey response rates: a review. J Comput-Mediat Commun.

[21] Lovato LC, Hill K, Hertert S, Hunninghake DB, Probstfield JL (1997). Recruitment for controlled clinical trials: literature summary and annotated bibliography. Control Clin Trials.

[22] Yammarino FJ, Skinner S, Childers TL (1991). Understanding mail survey response behavior. Pub Opinion Q.

[23] Horng S, Goss FR, Chen RS, Nathanson LA (2012). Prospective pilot study of a tablet computer in an emergency department. Int J Med Inform.

[24] Hopkins K, Gullickson A (1992). Response rates in survey research: a meat-analysis of the effects of monetary gratuities. J Exp Educ.

[25] Mapstone J, Elbourne D, Roberts I. Strategies to improve recruitment to research studies. Cochrane Database Syst Rev. 2007 Apr 18;(2):MR000013. 10.1002/14651858.MR000013.pub3 17443634

[26] Kotzee TJ, Couper ID (2006). What interventions do South African qualified doctors think will retain them in rural hospitals of the Limpopo province of South Africa?. Rural Remote Health.

[27] Bakker AB, Demerouti E (2007). The job demands-resources model: state of the art. J Manag Psychol.

[28] Rowe AK, de Savigny D, Lanata CF, Victora CG (2005). How can we achieve and maintain high-quality performance of health workers in low-resource settings?. Lancet.

[29] Coomarasamy A, Khan KS (2004). What is the evidence that postgraduate teaching in evidence based medicine changes anything?. Syst Rev Bmj.

[30] Howlett K (2010). Teen’s death ignites debate over emergency room closures. Globe Mail.

[31] Newman S (2008). Nelson doctors issue emergency aid plea. Globe Mail.

[32] Fleet R, Plant J, Ness R, Moola S (2013). Patient advocacy by rural emergency physicians after major service cuts: the case of Nelson, BC. Can J Rural Med.

[33] McGregor J, Hanlon N, Emmons S, Voaklander D, Kelly K (2005). If all ambulances could fly: putting provincial standards of emergency care access to the test in Northern British Columbia. Can J Rural Med.

[34] Rourke JT, Kennard M (2001). Emergency patient transfers from rural hospitals: a regional study. CJEM.

[35] Gauthier J, Hagerty J, Lamarche P, Lévesque JF, Morin D, Pineault R (2009). Entre adaptabilité et fragilité: les conditions d’accès aux services de santé des communautés rurales et éloignées.

[36] Hopkins S (1994). What is a Balint Group?. J Balint Soc.

[37] Russell DJ, Humphreys JS, McGrail MR, Cameron WI, Williams PJ (2013). The value of survival analyses for evidence-based rural medical workforce planning. Hum Resour Health.

